# Construction of a clinical prediction model for complicated appendicitis based on machine learning techniques

**DOI:** 10.1038/s41598-024-67453-4

**Published:** 2024-07-16

**Authors:** Wang Wei, Shen Tongping, Wang Jiaming

**Affiliations:** 1 The First Affiliated Hospital, Anhui University of Chinese Medicine, Hefei, China; 2 School of Information Engineering, Anhui University of Chinese Medicine, Hefei, China; 3 Graduate School, Angeles University Foundation, Angeles, Philippines

**Keywords:** Complicated appendicitis, Elderly, Machine learning, SHAP, Shiny, Cancer, Computational biology and bioinformatics, Diseases, Medical research

## Abstract

Acute appendicitis is a typical surgical emergency worldwide and one of the common causes of surgical acute abdomen in the elderly. Accurately diagnosing and differentiating acute appendicitis can assist clinicians in formulating a scientific and reasonable treatment plan and providing high-quality medical services for the elderly. In this study, we validated and analyzed the different performances of various machine learning models based on the analysis of clinical data, so as to construct a simple, fast, and accurate estimation method for the diagnosis of early acute appendicitis. The dataset of this paper was obtained from the medical data of elderly patients with acute appendicitis attending the First Affiliated Hospital of Anhui University of Chinese Medicine from January 2012 to January 2022, including 196 males (60.87%) and 126 females (39.13%), including 103 (31.99%) patients with complicated appendicitis and 219 (68.01%) patients with uncomplicated appendicitis. By comparing and analyzing the prediction results of the models implemented by nine different machine learning techniques (LR, CART, RF, SVM, Bayes, KNN, NN, FDA, and GBM), we found that the GBM algorithm gave the optimal results and that sensitivity, specificity, PPV, NPV, precision, recall, F1 and brier are 0.9167, 0.9739, 0.9429, 0.9613, 0.9429, 0.9167, 0.9296, and 0.05649, respectively. The GBM model prediction results are interpreted using the SHAP technology framework. Calibration and Decision curve analysis also show that the machine learning model proposed in this paper has some clinical and economic benefits. Finally, we developed the Shiny application for complicated appendicitis diagnosis to assist clinicians in quickly and effectively recognizing patients with complicated appendicitis (CA) and uncomplicated appendicitis (UA), and to formulate a more reasonable and scientific clinical plan for acute appendicitis patient population promptly.

## Introduction

The appendix is in the right lower abdomen, where food debris and bacteria accumulate and produce inflammation^1–3^. Acute appendicitis is the most common surgical emergency worldwide, with a lifetime risk of 7–8%^4^. The social impact and healthcare burden are very high^5^, and it is also one of the common causes of surgical acute abdomen in the elderly. Acute appendicitis can be categorized into two types: UA, which is defined as cellulitis appendicitis without signs of necrosis or perforation^6^. And CA, this has focal or transmural necrosis that may eventually lead to perforation^7^. It is essential to differentiate between these two conditions because UA can be treated conservatively with antibiotics without surgery^8,9^ and may even resolve independently without antibiotic therapy^10,11^. Patients with CA require emergency appendectomy.

Elderly people often suffer from a range of underlying diseases such as respiratory, circulatory, endocrine and metabolic disorders, and reduced immune function. Conservative treatment may also be attempted in elderly UA patients with mild clinical symptoms who have a strong desire to avoid surgery and accept the risk of recurrence. However, the probability of appendiceal perforation and death is significantly higher in elderly patients than in patients without perforation^12^. Moreover, delayed diagnosis of complicated appendicitis may lead to associated complications such as perforation and peritonitis, resulting in severe morbidity and mortality, especially in the elderly patient population with comorbidities^13^, as well as prolonged hospitalization, loss of employment, increased costs due to additional investigations, and psychosocial problems^14^.

Therefore, essential to diagnose acute appendicitis correctly, and a “two-stage” approach is generally used in clinical practice. The first stage involves diagnosing “acute appendicitis.” In patients who do not have acute appendicitis, the cause of the abdominal pain needs to be identified and treated promptly. After the diagnosis of acute appendicitis is confirmed, the second stage involves differentiating between UA and CA and adopting different treatment protocols for the other conditions.

Currently, historical information and laboratory findings are still considered the cornerstone of the diagnosis of acute appendicitis. Still, there is much intra-observer variability, and accuracy could be better. It has been shown that physicians fail to make a correct clinical diagnosis in all patients with acute abdominal pain based solely on history and routine laboratory tests^15^.

In addition to clinical tests, complete blood count parameters (leukocytes, neutrophils, lymphocytes, platelets, platelet derivatives), which are part of routine blood biochemistry parameters, as well as markers such as total bilirubin (TBil), C-reactive protein (CRP), and procalcitonin are widely used as a next step in the diagnosis of acute appendicitis, as they vary according to the presence and severity of inflammation^16^. Individually, these markers of inflammation have a weak discriminatory ability, but when combined, they have a higher discriminative ability in diagnosing acute appendicitis versus non-appendicitis^17^. However, according to a prospective data study including 1024 patients with clinical suspicion of acute appendicitis, this combination was found not to rule in or rule out appendicitis^18^ adequately.

Scoring systems such as AIRS and Alvarado, which consist of history, physical examination findings, blood biochemical parameters, and radiologic instruments and their combinations, can differentiate between simple and complex appendicitis^19^. None of these studies mentioned diagnostic accuracy measures, so sensitivity and specificity could not be calculated. Two other studies reported on the design of scoring systems, including clinical and biochemical features, neither reported diagnostic accuracy measures^20,21^. Imaging is essential in differentiating simple from complex appendicitis, with ultrasound and computed tomography (CT) imaging improving diagnostic sensitivity and specificity. Still, more sensitivity is needed for all parameters, while these tools have the disadvantages of being highly operator-dependent and radiation-exposed, respectively^22^.

The above suggests that the diagnosis of appendicitis relies on clinical evaluation, laboratory tests, and imaging studies, including ultrasound and computed tomography (CT) scans. Still, these methods are fraught with limitations, such as diagnostic inaccuracies and time-consuming procedures, which can lead to severe complications such as appendiceal perforation and sepsis.

To overcome these challenges, artificial intelligence (AI) has been widely used in clinical settings to assist doctors in medical diagnoses. Artificial intelligence refers to a machine’s ability to mimic human cognitive processes to perform tasks autonomously. Relevant literature has shown that AI techniques are advantageous in diagnosing acute appendicitis. Alramadhan et al. found that artificial neural networks (ANNs) can accurately predict the risk of intra-abdominal abscess (IAA) after appendectomy with an accuracy of 89.84%, sensitivity of 70%, and specificity of 93.61% on the test set^23^.

Xia et al. constructed a diagnostic model using an improved grasshopper optimization algorithm based support vector mechanism to distinguish between complex and uncomplicated appendicitis. The optimal model yielded an average of 83.56% accuracy, 81.71% sensitivity, 85.33% specificity, and 0.6732 Matthews correlation coefficient^24^. Kim et al. developed a model using multivariate logistic regression and Bayesian information criterion to assess the model’s performance in the validation dataset through calibration plots and area under the curve (AUC), respectively. The model’s calibration and discrimination performance could identify patients with definite uncomplicated appendicitis who could benefit from non-surgical treatment with a low risk of failure^25^.

Shahmoradi et al. compared the output of the optimized SVM artificial neural network with pathological findings. They showed that the network’s sensitivity, specificity, and accuracy for diagnosing acute appendicitis were 91.7%, 96.2%, and 95%, respectively^26^. Erkent et al. used a decision tree approach to determine the severity of acute appendicitis (AA) without imaging methods^27^.

The “Validity of machine learning in detecting complicated appendicitis in a resource-limited setting: findings from Vietnam” article also builds and validates a machine learning model to facilitate the detection of complex appendicitis^28^. Phan–Mai et al. used several machine learning methods, including SVM, to classify patients with complicated appendicitis and patients with uncomplicated appendicitis. The experimental results show that the GB model has high validity. Both articles are about detecting and determining complex appendicitis from a machine learning perspective. The differences are as follows: (1) The datasets are different; (2) The number of machine learning models used is different; (3) In our paper, after determining GB as the optimal model, we used SHAP technology to visualize the weights of each parameter; (4) We developed the complex appendix diagnostic Shiny app to diagnose UA and CA to maximize generalizability and facilitate translation into real-world clinical practice.

The clinical data for this study was obtained from the article “Development and Validation of a Clinical Prediction Model for Complicated Appendicitis in the Elderly”. The article used the SPSS 26. 0 and R 4.0.2 software to create a CA prediction model to help clinicians quickly determine the type of acute appendicitis. It was found that three parameters based on abdominal pain duration, peritonitis, and total bilirubin could help physicians quickly and effectively determine UA or CA. we re-examined the study based on the clinical data provided in this article using machine learning methods. We used nine machine methods to construct the most suitable clinical prediction model and further demonstrated the importance of each parameter using SHAP technique. Finally we develop the Shiny application for complicated appendicitis diagnosis to assist clinicians in quickly and effectively recognizing patients with CA and UA.

Unlike traditional medical statistical methods, machine learning techniques predict new observations by learning based on existing data. However, a significant problem with many state-of-the-art machine learning models is the need for more transparency and interpretability. To be able to interpret the results of predictions and judgments of machine learning models, explainable artificial intelligence (XAI) techniques are applied in clinical research, among which the SHAP technique is one of the methods of XAI, which determines values showing the direction and magnitude of the contribution of a variable to the estimation of an ML model and provides a visualization of the variable's contribution^30^.

In this study, we predicted UA and CA by ML modeling using patients’ clinical and biochemical examination indexes and interpreted the model results using SHAP technique. The main findings and contributions of this paper are as follows:

(1) ML models were created to predict patients with UA and CA accurately.

(2) The GBM model performed well in differentiating patients.

(3) To explain the model, we utilized SHAP technique to show the importance of different parameters. And we have developed CA diagnosis Shiny application which helps clinicians to diagnose UA and CA.

## Materials and methods

### Study population

The data on rectal cancer in the elderly used in this paper were obtained from the article “Development and Validation of a Clinical Prediction Model for Complicated Appendicitis in the Elderly”^29^. The dataset collected clinical data of elderly patients with acute appendicitis who attended the First Affiliated Hospital of Anhui University of Chinese Medicine from January 2012 to January 2022. Inclusion criteria: (1) age more than 65 years; (2) pathological diagnosis of acute appendicitis after surgery; (3) no life-threatening cardiovascular or cerebrovascular diseases at the time of admission. Exclusion criteria: (1) age less than 65 years; (2) periappendiceal abscess; (3) combination of significant traumas and surgical histories; (4) combination of hematological disorders, malignant neoplasms, and psychiatric disorders. This study was a secondary analysis of retrospective data and was a retrospective, multi-cohort, observational study using de-identified data. Therefore, consent and research ethics committee approval was not required.

### Data set indicators and grouping

In this study, data on patients’ basic conditions, medical history and laboratory findings were collected by processing their chart record forms, and 30 clinical indicators were finalized, namely: age, gender, history of diabetes mellitus, duration of abdominal pain (APD), severe pain in the right lower abdomen, Nausea or vomiting, body temperature (TEMP), history of appendicitis, history of peritonitis, white blood cell count (WBC), neutrophil percentage (NEUT%), neutrophil count (NEUT), lymphocyte percentage (LY%), lymphocyte count (LY), neutrophil-to-lymphocyte ratio (NLR), neutrophil-to-lymphocyte ratio (NLR), monocyte percentage, monocyte count, red blood cell count (RBC), hematocrit, hemoglobin (HGB), platelets (PLT), prothrombin time (PT), activated partial thromboplastin time (APTT), alanine aminotransferase (ALT), aspartate aminotransferase (AST), total bilirubin (TBil), creatinine (Cr), and Ca^2+^.

### Model development and validation

The dataset was randomly divided into 70% for the training set and 30% for the testing set. We used nine machine learning algorithms, such as LR, CART, RF, SVM, Bayes, KNN, NN, FDA, and GBM, to make predictions and judgments on complex appendiceal clinical data, and each model was cross-validated by five tenfold and optimal settings of model parameters. We then assessed the performance of the models in the test set using the machine learning evaluation metrics accuracy (95% CI), sensitivity, specificity, PPV, NPV, precision, recall, F1, and brie scorer. We also plot calibration and DCA curves for each model to validate the model performance and clinical benefit curves further.

### Statistical methods

All analyses in this paper mainly used R software (4.3.0) and Python software (3.11.0). Firstly, 12-factor variables with clinical significance were screened by unifactorial and multifactorial analyses, which were CA, nausea_or_vomiting, peritonitis, periappendiceal_fat_infiltration, TEMP, APD, WBC, NEUT, LY, NLR, AST, and Tbil, using these variables as parameters for the nine machine learning models. The method of the LR model is “glm”, CART model is “rpart”, RF model is “rf”, SVM model is “svmLinear3”, Bayes model is “bayesglm”, KNN model is “knn”, NN model is “nnet”, FDA model is “fda”, GBM model is “gbm”.

We plotted baseline tables and ROC curves using the packages plotROC, caret, autoReg, pROC, and e1071 in the R software. We visualized the parameters in the models by drawing SHAP plots using Python software. Bilateral P < 0.05 was considered statistically significant.

### SHAP

**Figure 1:**
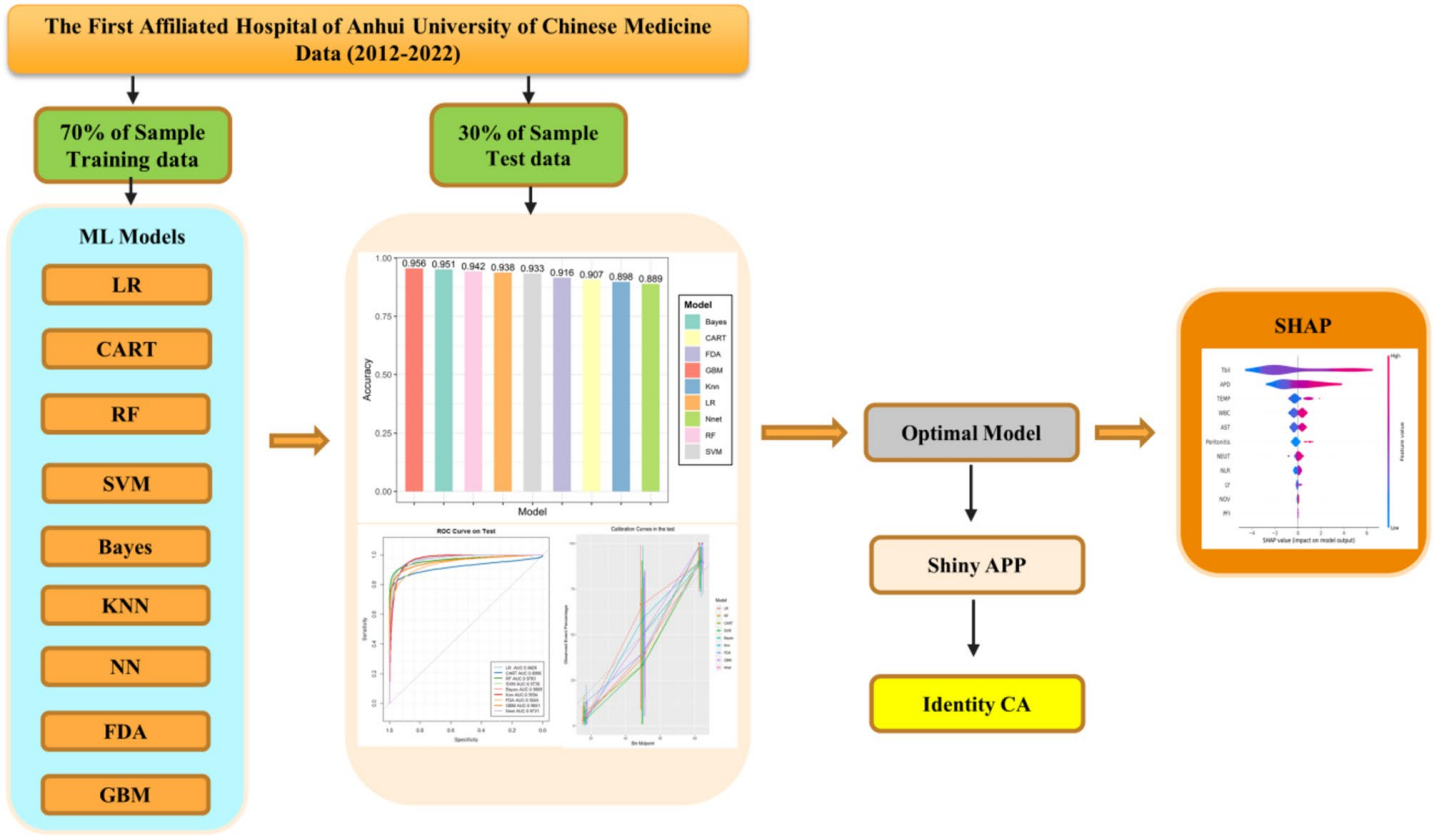
Technical flowchart of the article.

To explain the contribution of each independent variable in a machine learning model to the model’s results, this paper uses the SHAP technique, which is based on the optimal Shapley value for the method of interpreting the individual and global predictions of the model. The Shapley value considers all the potential estimates of an observation using all possible combinations of variables. In this case, the purpose of SHAP is to explain the estimation of any observation by calculating the contribution of each variable to the estimation. The flowchart of all the technical methods used in this study is shown in Fig. 1.

### Approval for human experiments

All experiments were performed in accordance with relevant named guidelines and regulations.

## Results

### Table of patients’ baseline characteristics

Three hundred twenty-two patients were included in this study, of whom 126 were female and 196 were male. The mean age of all patients was 71.59 ± 4.71 years, and the characteristics of the included patients are shown in Table 1.

**Table 1:** Table of baseline patient characteristics.

Characteristics	Variables	UA (N = 219)	CA (N = 103)	OR (univariable)	OR (multivariable)
Gender	0	90 (41.1%)	36 (35%)		
	1	129 (58.9%)	67 (65%)	1.30 (0.80–2.11, P = 0.292)	
History_of_diabetes	0	156 (71.2%)	82 (79.6%)		
	1	63 (28.8%)	21 (20.4%)	0.63 (0.36–1.11, P = 0.112)	
Shifiting_pain	0	102 (46.6%)	31 (30.1%)		
	1	117 (53.4%)	72 (69.9%)	2.02 (1.23–3.33, P = 0.005)	1.02 (0.25–4.25, P = 0.976)
Nausea_or_vomiting	0	118 (53.9%)	36 (35%)		
	1	101 (46.1%)	67 (65%)	2.17 (1.34–3.53, P = 0.002)	2.29 (0.60–8.77, P = 0.226)
History_of_appendicitis	0	198 (90.4%)	90 (87.4%)		
	1	21 (9.6%)	13 (12.6%)	1.36 (0.65–2.84, P = 0.410)	
Peritonitis	0	203 (92.7%)	62 (60.2%)		
	1	16 (7.3%)	41 (39.8%)	8.39 (4.41–15.97, P < 0.001)	10.26 (1.08–97.24, P = 0.042)
Appendix_bezoar	0	138 (63%)	63 (61.2%)		
	1	81 (37%)	40 (38.8%)	1.08 (0.67–1.75, P = 0.749)	
Periappendiceal_fat_infiltration	0	180 (82.2%)	60 (58.3%)		
	1	39 (17.8%)	43 (41.7%)	3.31 (1.96–5.58, P < 0.001)	1.82 (0.40–8.27, P = 0.441)
TEMP	Mean ± SD	37.2 ± 0.5	37.7 ± 0.9	2.58 (1.79–3.73, P < 0.001)	2.16 (0.85–5.48, P = 0.107)
Age	Mean ± SD	71.3 ± 4.8	72.1 ± 4.5	1.04 (0.99–1.09, P = 0.158)	
APD	Mean ± SD	30.3 ± 16.8	50.8 ± 13.4	1.08 (1.06–1.10, P < 0.001)	1.10 (1.06–1.15, P < 0.001)
WBC	Mean ± SD	12.5 ± 3.3	14.6 ± 3.7	1.20 (1.11–1.29, P < 0.001)	1.01 (0.77–1.32, P = 0.942)
Percentage.of.NEUT	Mean ± SD	80.0 ± 7.6	84.3 ± 6.2	1.10 (1.06–1.14, P < 0.001)	1.08 (0.96–1.22, P = 0.219)
NEUT	Mean ± SD	11.2 ± 3.8	12.8 ± 3.2	1.13 (1.06–1.21, P < 0.001)	1.27 (0.94–1.70, P = 0.116)
Percentage.of.LY	Mean ± SD	11.6 ± 6.4	10.2 ± 4.5	0.96 (0.92–1.00, P = 0.044)	0.91 (0.80–1.03, P = 0.134)
LY	Mean ± SD	1.6 ± 0.7	1.5 ± 0.7	0.66 (0.46–0.96, P = 0.028)	1.47 (0.38–5.66, P = 0.579)
NLR	Mean ± SD	8.1 ± 5.0	10.6 ± 5.5	1.09 (1.04–1.14, P < 0.001)	1.04 (0.85–1.26, P = 0.721)
Percentage_of_monocytes	Mean ± SD	5.5 ± 1.9	5.2 ± 2.0	0.92 (0.81–1.04, P = 0.193)	
Monocytes	Mean ± SD	0.7 ± 0.4	0.7 ± 0.3	0.67 (0.36–1.26, P = 0.214)	
RBC	Mean ± SD	5.0 ± 0.6	5.0 ± 0.7	1.07 (0.75–1.53, P = 0.697)	
Hematocrit	Mean ± SD	41.1 ± 4.4	41.1 ± 4.4	1.00 (0.94–1.05, P = 0.908)	
HGB	Mean ± SD	122.0 ± 21.8	120.8 ± 21.3	1.00 (0.99–1.01, P = 0.654)	
PLT	Mean ± SD	234.3 ± 56.7	234.8 ± 63.8	1.00 (1.00–1.00, P = 0.942)	
PT	Mean ± SD	12.4 ± 1.5	12.7 ± 1.8	1.11 (0.96–1.29, P = 0.143)	
APTT	Mean ± SD	31.3 ± 3.3	31.0 ± 3.1	0.98 (0.91–1.05, P = 0.536)	
ALT	Mean ± SD	31.0 ± 12.1	34.6 ± 17.1	1.02 (1.00–1.04, P = 0.034)	1.04 (0.99–1.10, P = 0.126)
AST	Mean ± SD	26.1 ± 12.4	31.7 ± 14.1	1.03 (1.01–1.05, P < 0.001)	1.07 (1.01–1.13, P = 0.024)
Tbil	Mean ± SD	14.2 ± 3.6	24.1 ± 4.5	1.86 (1.61–2.14, P < 0.001)	2.14 (1.63–2.83, P < 0.001)
Cr	Mean ± SD	89.1 ± 17.7	90.0 ± 22.3	1.00 (0.99–1.01, P = 0.666)	
Ca^2+^	Mean ± SD	2.3 ± 0.1	2.3 ± 0.1	0.21 (0.02–1.76, P = 0.151)	

There was no significant difference in age between the CA group and the UA group (72.1 ± 4.5 vs. 71.3 ± 4.8 years, P = 0.157), the temperature in the CA group was higher than that in the UA group (37.7 ± 0.9 vs. 37.2 ± 0.5, P < 0.001), and APD was significantly higher in the CA group than in the UA group (50.8 ± 13.4 vs. 30.3 ± 16.8, P < 0.001). In univariate analysis, nausea_or_vomiting, peritonitis, periappendiceal_fat_infiltration, TEMP, APD, WBC, Percentage.of.NEUT, NEUT, Percentage.of.LY, LY, NLR, ALT, AST, Tbil, and 14 other factors were statistically significant at P < 0.05. We included variables with P < 0.05 in the univariate logistic regression analysis into the multivariate logistic regression, and the factors peritonitis, APD, AST, and Tbil were statistically significant at P < 0.05. After careful consideration, we finally used the variables CA, nausea_or_vomiting, peritonitis, periappendiceal_fat_infiltration, TEMP, APD, WBC, NEUT, LY, NLR, AST, and Tbil as the parameters of the nine machine learning models.

### Comparative performance analysis of ML models for CA recognition

**Figure 2:**
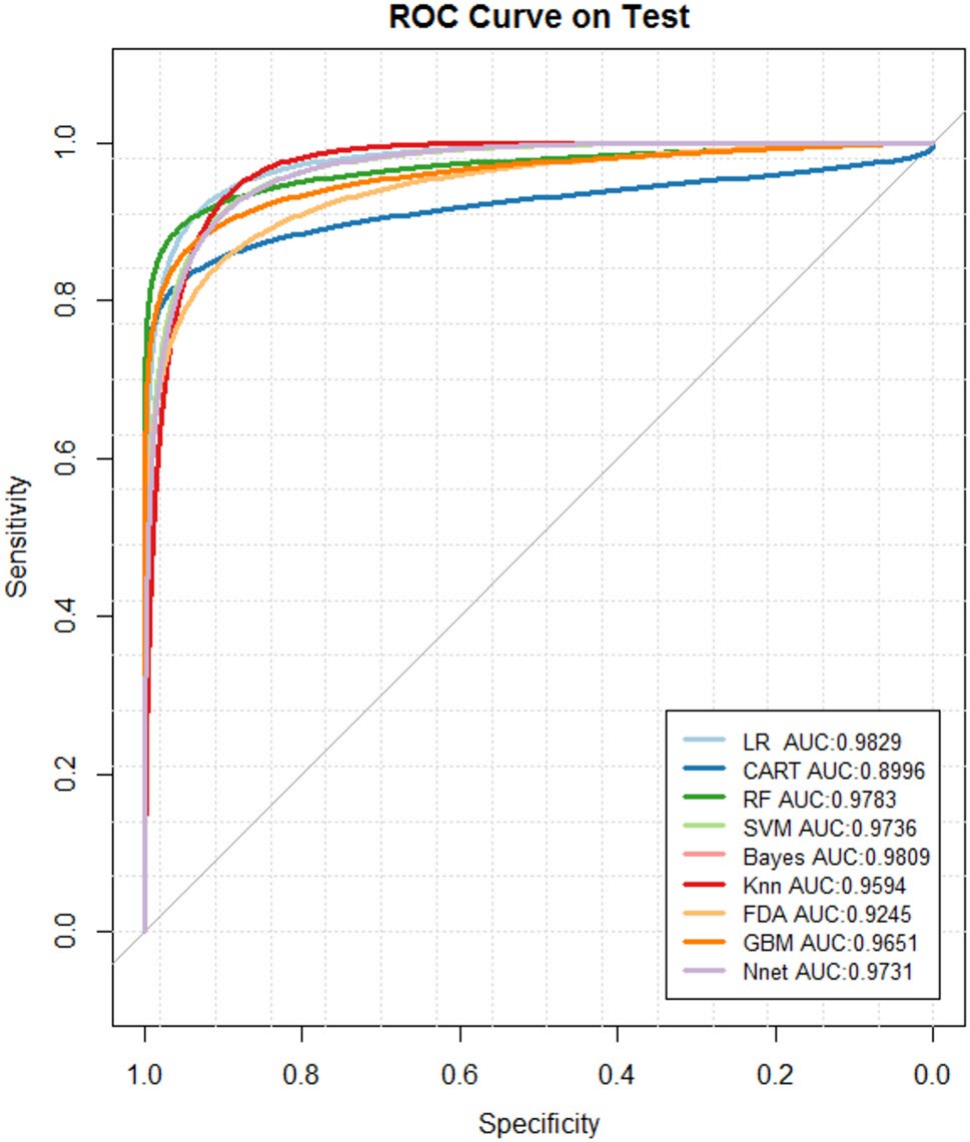
ROC curves for nine machine learning models.

**Figure 3:**
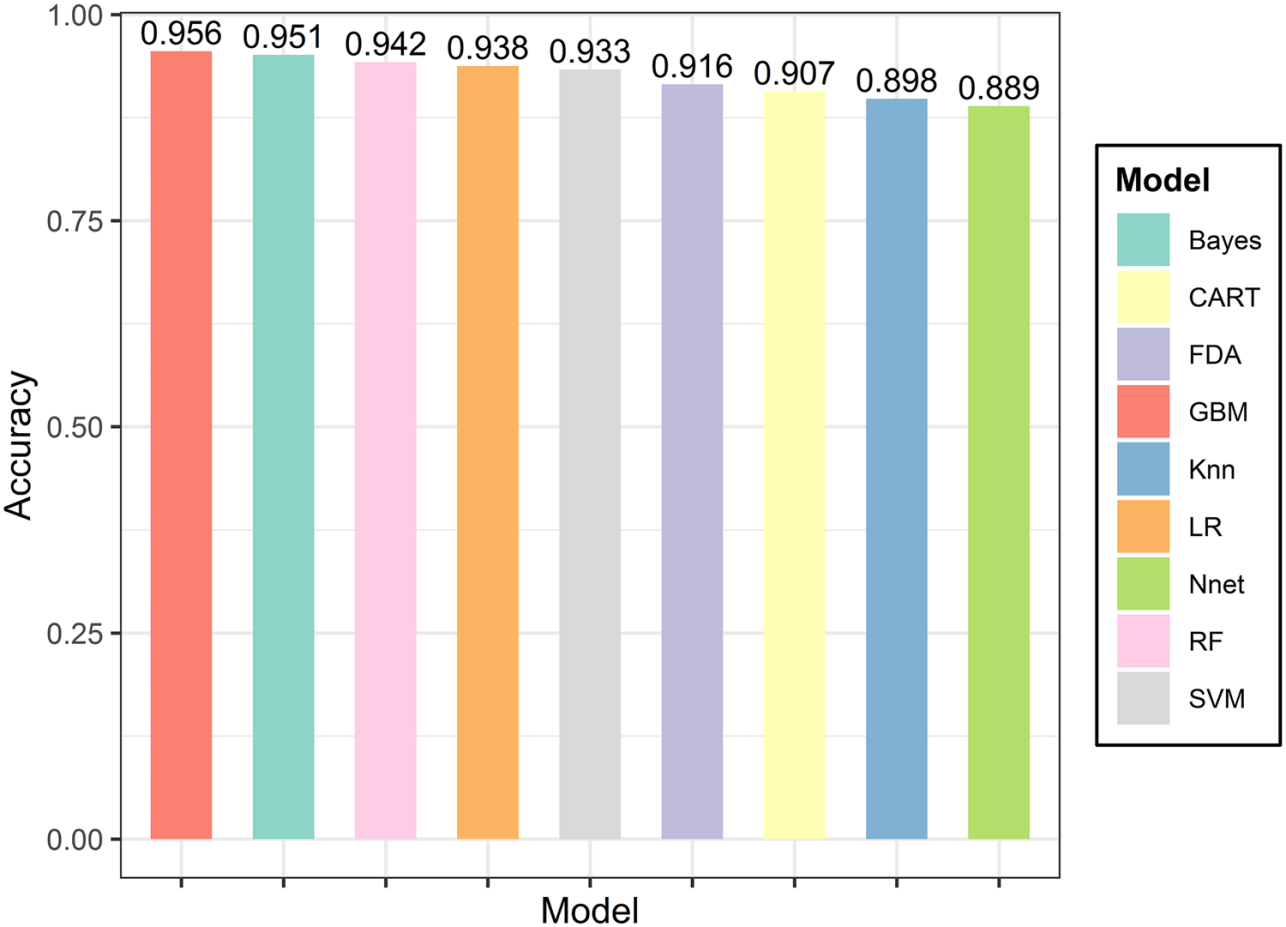
Accuracy plot of nine machine learning models.

We applied nine machine learning models to the training set, and the performance of each model is shown in Table 2. The AUC of the nine models ranges from 0.8996 to 0.9829, and the receiver operating characteristic curve (ROC) are shown in Fig. 2. We found that GBM had the highest accuracy (accuracy: 0.9556; CI 0.9198–0.9785), and the accuracy of the other eight machine learning models ranged from 0.8889 to 0.9511. The comparison of the individual models’ accuracy is shown in Fig. 3.

**Table 2:** Results of nine machine learning models.

Model	Accuracy (95% CI)	Sensitivity	Specificity	PPV	NPV	Precision	Recall	F1	Brier
LR	0.9378 (0.8978, 0.9656)	0.8333	**0.9869**	**0.9677**	0.9264	**0.9677**	0.8333	0.8955	0.05139
CART	0.9067 (0.8609, 0.9413)	**0.9167**	0.9020	0.8148	0.9583	0.8148	**0.9167**	0.8627	0.07760
RF	0.9422 (0.9032, 0.9689)	0.8611	0.9804	0.9538	0.9375	0.9538	0.8611	0.9051	0.05199
SVM	0.9333 (0.8924, 0.9622)	0.8194	0.9869	0.9672	0.9207	0.9672	0.8194	0.8872	0.05678
Bayes	0.9511 (0.9142, 0.9753)	0.8750	0.9869	0.9692	0.9437	0.9692	0.8750	0.9197	**0.05087**
KNN	0.8978 (0.8506, 0.9341)	0.8056	0.9412	0.8657	0.9114	0.8657	0.8056	0.8345	0.06556
NN	0.8889 (0.8404, 0.9268)	0.7500	0.9542	0.8852	0.8902	0.8852	0.7500	0.8120	0.06524
FDA	0.9156 (0.8713, 0.9484)	0.8194	0.9608	0.9077	0.9187	0.9077	0.8194	0.8613	0.09106
GBM	**0.9556 (0.9198, 0.9785)**	**0.9167**	0.9739	0.9429	**0.9613**	0.9429	**0.9167**	**0.9296**	0.05649

Significant values are given in bold.

The authors compared the prediction performance of the nine ML algorithms in terms of multiple metrics. The authors found that GBM has the best accuracy, sensitivity, NPV, recall, and F1 among all ML algorithms; LR has the best specificity, PPV, and precision.

The other seven machine learning algorithms also achieved good prediction scores. We also evaluated the accuracy of different models by calculating brier scores. Bayes, LR, RF, SVM, and GBM have better reliability assessments of brier scores than CART, KNN, NN, and FDA.

Finally, by comprehensively analyzing the prediction performance of different models, we found that GBM has the highest accuracy and robustness in identifying CAs. Therefore, the GBM model was selected for subsequent analysis.

### ML model calibration curve and DCA curve

We further validated that the model used in this paper can be applied in the clinic through calibration and decision curve analysis.

**Figure 4:**
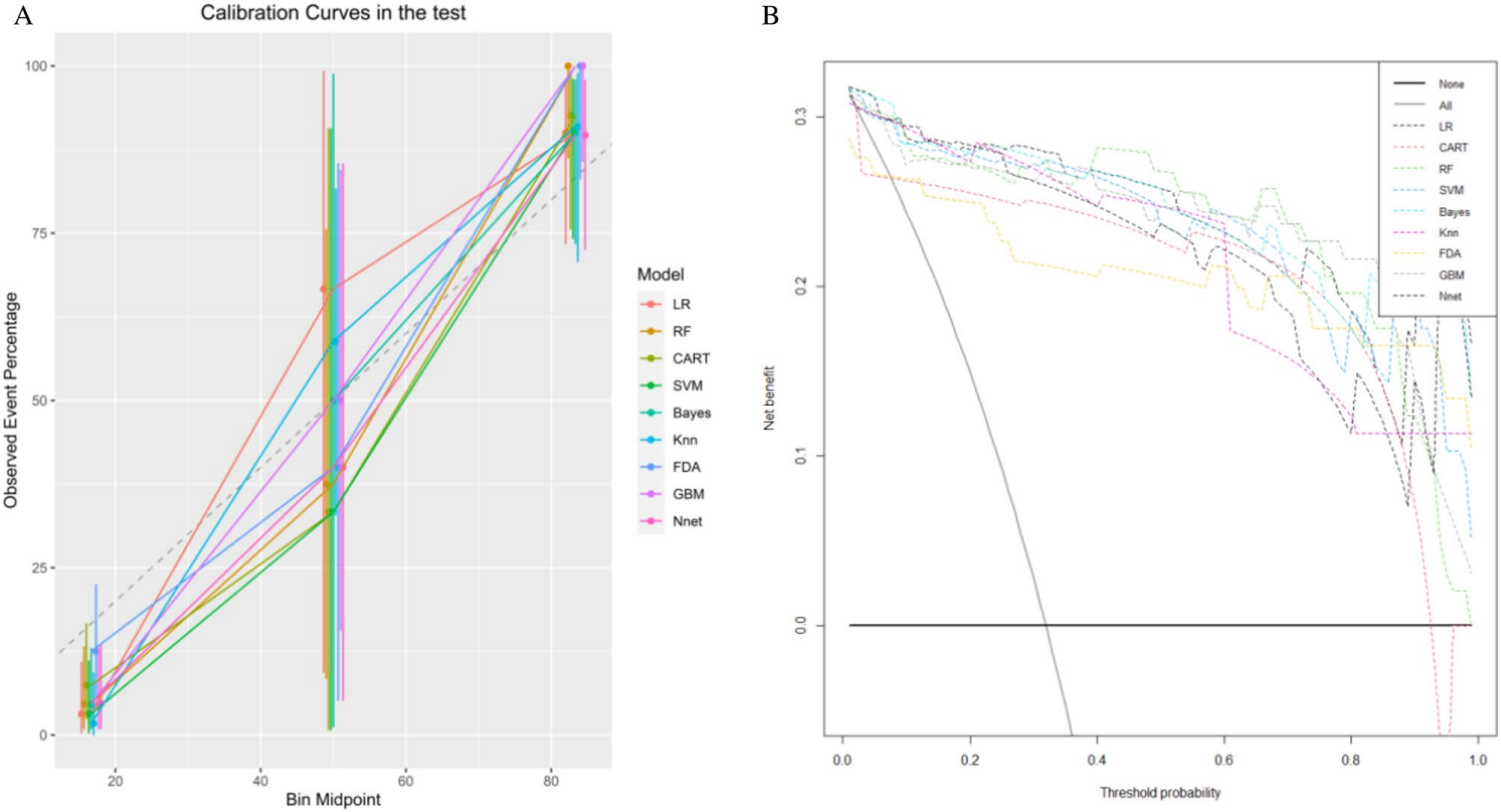
(**A**) Calibration curves. (**B**). DCA curves.

The calibration curve is primarily used to measure the accuracy of the model's predictions; the closer the curve is to the middle diagonal, the better the model performs. Figure 4A shows that the GBM model has the best performance of the calibration curve, which is more predictive than the other eight machine learning models.

Traditional machine learning evaluation metrics, which are mainly used to predict the diagnostic accuracy of a model, fail to consider the clinical utility of a particular model. DCA can integrate patient or decision-maker preferences into the analysis to assess the clinical benefit, which has some clinical utility.

Figure 4B shows that all nine machine learning models have certain clinical gains, among which the GBM, the FDA, and the KNN models achieve the best clinical gains.

### Model interpretation and individual analysis

**Figure 5:**
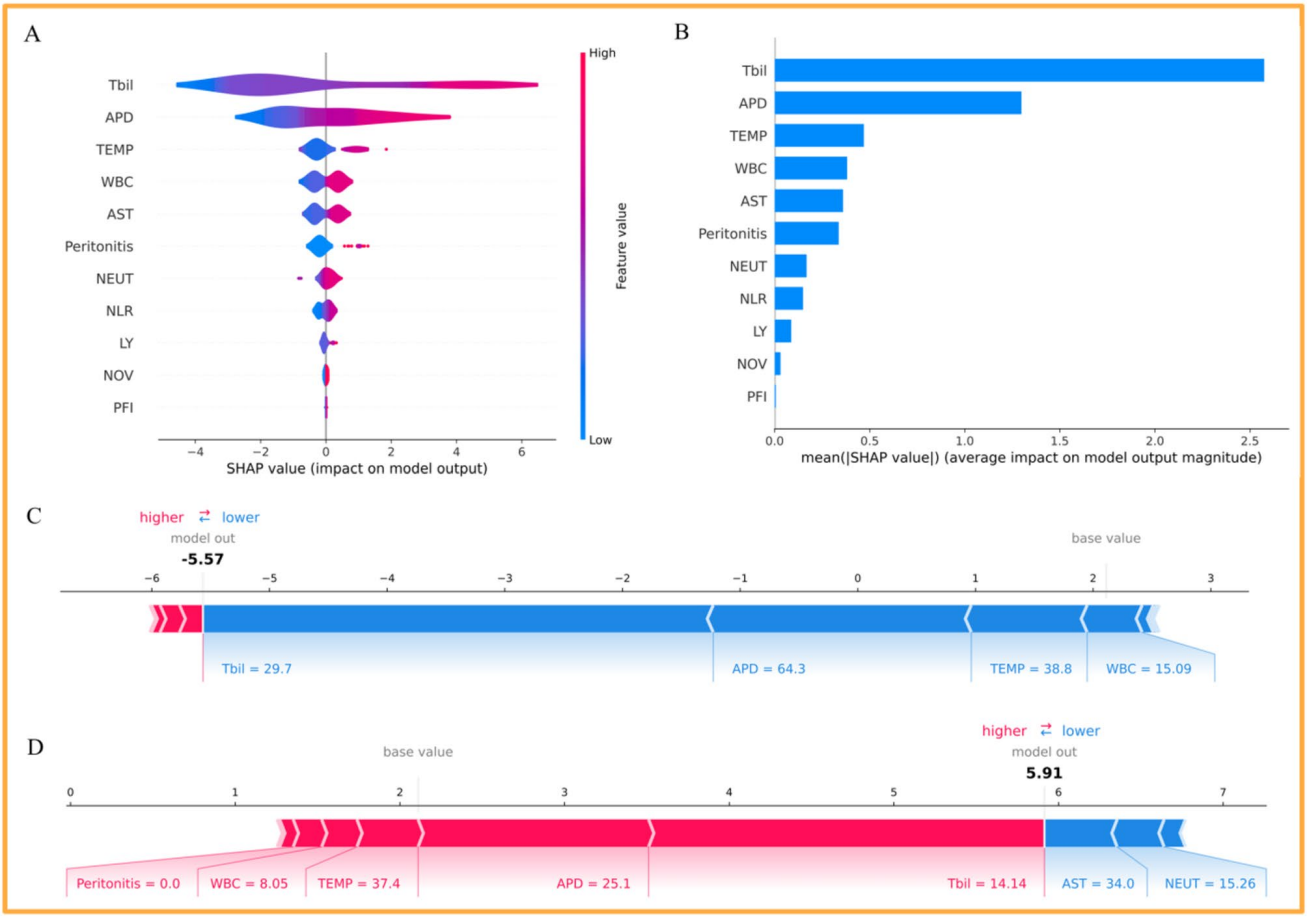
The visual representation of the GBM model.

The authors applied SHAP to interpret the effect of the specified features in the GBM model on CA. Figure 5A shows the top 11 predictors of complex appendicitis. These include Tbil, APD, TEMP, WBC, AST, Peritonitis, NEUT, NLR, LY, NOV, and PFI; these variables' mean significance is shown in Fig. 5B.

Figure 5C, D shows the SHAP plots for two patients (a patient with CA and a patient with UA) to illustrate the interpretability of the model. The CA patient had an elevated Tbil value, a greater APD index, an elevated body temperature, and a greater WBC index, and based on this information, the model predicted a decreased risk for the complex rectal cancer patient. The UA patient had a decreased Tbil value, a lower APD, a lower body temperature, a decreased WBC index, a lower AST value, and a higher NEUT, and the model predicted an increase in the risk of the patient becoming a CA patient.

## Discussion

The clinical manifestations of UA and CA are similar and not easy to distinguish. Elderly patient groups are pretty resistant to surgical treatment due to their unique physical and physiological states. However, once CA is diagnosed, surgical treatment should be carried out immediately; otherwise, it will be life-threatening. Therefore, how to predict CA in elderly patients with appendicitis early, quickly and accurately so as to guide the clinical individualized treatment of elderly patients with appendicitis has been a difficult hotspot in clinical research. With the development of precision medicine and individualized medicine, prediction models based on artificial intelligence technology have gradually become a hot spot of clinical research in recent years.

This study constructed a clinical prediction model based on machine learning technology by retrospectively analyzing the clinical data of elderly appendicitis patients. We also conduct comparative analysis and internal validation boarding of different machine models to deeply explore the clinical practicability of the constructed prediction model, realize early assessment and individualized treatment of elderly patients with appendicitis, and help clinicians make more scientific clinical treatment decisions.

The total of nine machine learning algorithms were used in this study, and ML methods, including LR, CART, RF, SVM, Bayes, KNN, NN, FDA, and GBM, had excellent performance in classifying patients with CA and UA. By comprehensively analyzing the predictive performance of the different models, we found that GBM had the highest accuracy and robustness in identifying CA, and GBM had the highest accuracy (accuracy: 0.9556; CI 0.9198–0.9785), which indicated that the model had the excellent predictive ability for CA, and thus the GBM model was selected for subsequent analysis. The calibration plot showed that the GBM model prediction curve agreed with the observed curve. The DCA plot showed that using the GBM model, the FDA model, and the KNN to predict and identify CA and take appropriate therapeutic measures could benefit patients in clinical practice. Finally, the SHAP technique was used to interpret the effects of the specified features in the GBM model on CA, and it was found that three indicators, including Tbil, APD, and TEMP, had the most significant impact on predicting CA.

Relevant literature studies have found that *Escherichia coli* is the main causative microorganism of appendicitis, causing dose-dependent cholestasis and erythrocyte hemolysis, which increases the bilirubin load^30,31^. Severe inflammation in patients with CA can lead to intestinal edema and decreased peristalsis, leading to cholestasis^32^. Some studies in the literature have found that Tbil values above 1.0 mg/dL, gangrenous or perforated appendicitis are associated and that patients with gangrenous perforated appendicitis have a higher incidence of hyperbilirubinemia than patients with acute uncomplicated appendicitis^33^. Relevant literature has shown that body temperature above 37.3 °C significantly differentiates patients with simple and complicated appendicitis^34–37^. Therefore, it is essential to focus on elderly patients when their body temperature exceeds normal levels. Studies have shown that pre-hospitalization APD in elderly patients with acute appendicitis is the most critical risk factor for perforation, with a 9% increase in the relative risk of perforation for each day of delay^38,39^. Studies of CA patients in low- and middle-income countries have also shown that the longer the APD, the higher the incidence of CA, and surgeons should be more aware of the importance of APD in elderly patients^40,41^.

**Figure 6:**
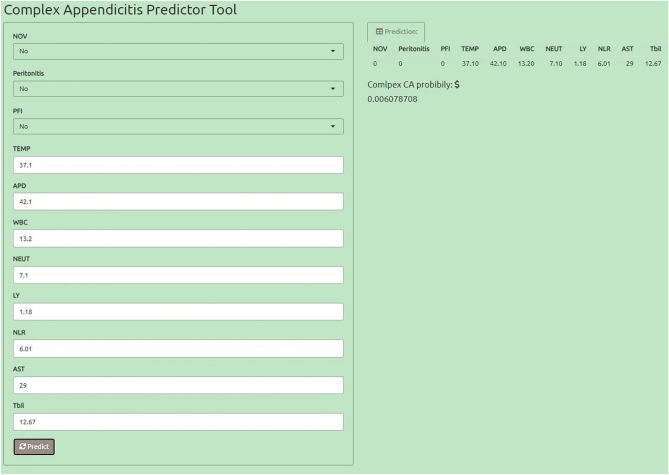
Shiny application diagnosed as UA.

**Figure 7:**
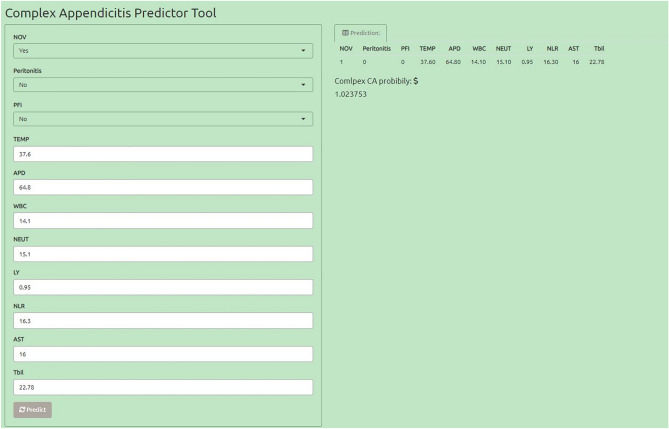
Shiny application diagnosed as CA.

We developed the complex appendix diagnostic Shiny app to diagnose UA and CA to maximize generalizability and facilitate translation into real-world clinical practice. The results shown in Figs. 6, 7 allow clinicians to calculate the individualized risk of a complex appendix based on six authoritative diagnostic guidelines. Available from: https://medicalpredictor.shinyapps.io/medic_predict/.

In actual clinical practice, the model should be viewed as a whole rather than making diagnostic predictions based on individual features without considering the remaining vital features. This means that the model needs the panel’s inputs to distinguish between UA and CA’s clinical data features.

From a broader perspective, almost all of our results are similar to recent medical literature published worldwide. The consistency of our findings demonstrates the clinical relevance of our model.

Our study has some limitations. First, the sample size of such studies is relatively small, and the data were collected from a single hospital. This limits the generalizability of our research. Further studies are needed to confirm our findings. Moreover, the incidence of complex appendicitis cases was relatively low, and the data was not as qualitative as expected. Although our model showed favorable results, APD, temperature, and Tbil were independent predictors of CA in elderly patients. Clinical prediction models constructed based on these three indicators can predict CA in elderly patients with high accuracy, which can help clinicians develop more rational clinical protocols, thus saving medical costs and improving patient prognosis. However, they should be used as screening tools, and experts and other relevant clinical evidence still need to decide the actual diagnosis.

## Conclusion

In this paper, we have explored the value of machine learning techniques in clinical prediction using a total of nine machine learning algorithms such as LR, CART, RF, SVM, Bayes, KNN, NN, FDA, and GBM to differentiate between patients with UA and patients with CA, and found that the machine learning algorithms have good performance in classifying appendicitis, with GBM providing the highest level of accuracy in recognizing CA and the highest level of robustness. We also developed the Shiny application for complex appendiceal diagnosis to assist clinicians in developing more rational clinical protocols, thereby saving healthcare costs and improving patient prognosis.

## Data Availability

Full collected data can be obtained through email from the corresponding author.

## Abbreviations

APD: Abdominal pain duration
TEMP: Temperature
WBC: White blood cell count
NEUT%: Neutrophil percentage
NEUT: Neutrophil count
LY%: Lymphocyte percentage
LY: Lymphocyte count
NLR: Neutrophil-to-lymphocyte ratio
RBC: Red blood cell count
HGB: Hematocrit, hemoglobin
PLT: Platelets
PT: Prothrombin time
APTT: Activated partial thromboplastin time
ALT: Alanine aminotransferase
AST: Aspartate aminotransferase
TBil: Total bilirubin
Cr: Creatinine
